# Functionalization of MXene Nanosheets for Polystyrene towards High Thermal Stability and Flame Retardant Properties

**DOI:** 10.3390/polym11060976

**Published:** 2019-06-03

**Authors:** Jing-Yu Si, Benjamin Tawiah, Wei-Long Sun, Bo Lin, Cheng Wang, Anthony Chun Yin Yuen, Bin Yu, Ao Li, Wei Yang, Hong-Dian Lu, Qing Nian Chan, Guan Heng Yeoh

**Affiliations:** 1 Department of Chemical and Materials Engineering, Hefei University, 99 Jinxiu Avenue, Hefei, Anhui 230601, China; sijyo@hfuu.edu.cn (J.-Y.S.); 18256858944@163.com (W.-L.S.); luhdo@hfuu.edu.cn (H.-D.L.); 2 Institute of Textiles & Clothing, The Hong Kong Polytechnic University, Hung Hom, Hong Kong; benjamin.tawiah@connect.polyu.hk; 3 School of Mechanical and Manufacturing Engineering, University of New South Wales, Sydney, NSW 2052, Australia; bo.lin@unsw.edu.au (B.L.); c.wang@unsw.edu.au (C.W.); c.y.yuen@unsw.edu.au (A.C.Y.Y.); ao.li@unsw.edu.au (A.L.); qing.chan@unsw.edu.au (Q.N.C.); g.yeoh@unsw.edu.au (G.H.Y.); 4 Department of Architecture and Civil Engineering, City University of Hong Kong, Tat Chee Avenue, Kowloon, Hong Kong

**Keywords:** MXene, functionalization, polystyrene, thermal stability, flame retardant

## Abstract

Fabricating high-performance MXene-based polymer nanocomposites is a huge challenge because of the poor dispersion and interfacial interaction of MXene nanosheets in the polymer matrix. To address the issue, MXene nanosheets were successfully exfoliated and subsequently modified by long-chain cationic agents with different chain lengths, i.e., decyltrimethylammonium bromide (DTAB), octadecyltrimethylammonium bromide (OTAB), and dihexadecyldimethylammonium bromide (DDAB). With the long-chain groups on their surface, modified Ti_3_C_2_ (MXene) nanosheets were well dispersed in *N*,*N*-dimethylformamide (DMF), resulting in the formation of uniform dispersion and strong interfacial adhesion within a polystyrene (PS) matrix. The thermal stability properties of cationic modified Ti_3_C_2_/PS nanocomposites were improved considerably with the temperatures at 5% weight loss increasing by 20 °C for DTAB-Ti_3_C_2_/PS, 25 °C for OTAB-Ti_3_C_2_/PS and 23 °C for DDAB-Ti_3_C_2_/PS, respectively. The modified MXene nanosheets also enhanced the flame-retardant properties of PS. Compared to neat PS, the peak heat release rate (PHRR) was reduced by approximately 26.4%, 21.5% and 20.8% for PS/OTAB-Ti_3_C_2_, PS/DDAB-Ti_3_C_2_ and PS/DTAB-Ti_3_C_2_, respectively. Significant reductions in CO and CO_2_ productions were also obtained in the cone calorimeter test and generally lower pyrolysis volatile products were recorded by PS/OTAB-Ti_3_C_2_ compared to pristine PS. These property enhancements of PS nanocomposites are attributed to the superior dispersion, catalytic and barrier effects of Ti_3_C_2_ nanosheets.

## 1. Introduction

Polystyrene (PS) is widely used in many fields due to its low density, excellent chemical resistance, and ease of processing [1,2,3]. However, with the intrinsic high flammability, its application range is limited. Therefore, it is imperative to improve the flame retardancy of PS where high fire safety standards are required. To date, many efforts have been made to improve the flame retardancy of PS. Reactive flame retardants (FRs) containing phosphorus and nitrogen used in PS have been explored [4,5]. Unfortunately, the flame-retardant PS usually possesses limited flame retardancy, owing to the low concentration of FRs while retaining the physical properties of PS. For example, Tai et al. synthesized a PS copolymer modified with a phosphorus-containing monomer, acryloxyethyl phenoxy phosphorodiethyl amidate (AEPPA) by free radical bulk polymerization [5]. The micro combustion calorimeter results indicated that AEPPA can decrease the peak heat release rate (approximately a 22.4% decrease) and the heat release capacity (approximately a 22.1% decrease). However, the glass transition temperature and initial degradation temperature of the copolymers were reduced with the increase of AEPPA loading. Another common approach to endow PS with flame retardancy is to incorporate addition-type additives, such as magnesium hydroxide [6], ammonium polyphosphate [7] or aluminium hypophosphite [8]. This method can endow flame retardancy; for instance, the peak heat release rate (PHRR) of the PS composites can be decreased (by approximately 83.6%) by adding 30 wt.% aluminium hypophosphite [8]. However, there are still many issues associated with the utilization of this approach, such as reduced mechanical properties due to poor compatibility and low flame retardancy durability because of the leaching of FR from PS. Despite high efficiency on PS, halogenated flame retardants are being phased out due to their toxic and corrosive nature upon burning.

In recent years, nanofillers, including polyhedral oligomeric silsesquioxane (POSS) [9,10,11], fullerene [12,13] and carbon nanotubes (CNTs) [14,15,16], have been shown to significantly improve the thermal, mechanical and flame-retardant properties of polymers at very small loading concentrations. Among various nanofillers, two-dimensional (2D) nanomaterials have been revealed to impart excellent flame retardancy to polymeric materials by the so-called “tortuous path” barrier effect which inhibits heat and mass exchange between the gas phase and the solid phase [17]. Two-dimensional nanomaterials, such as nanoclay [18,19,20], layered double hydroxides [21,22], graphene [23,24,25,26], transition metal disulfides [27,28], graphitized carbon nitride [29] and hexagonal boron nitride [30,31,32] have exhibited promising flame-retardant improvement for polymeric materials in recent years.

Recently, a new emerging 2D material called transition metal carbides/nitrides (MXenes) have gained extensive attention from the academic community, due to their high metallic conductivity [33] and tunable surface functional groups [34]. Versatile applications of MXenes in supercapacitors, batteries and fuel cells [35], chemical sensors [36] and catalysts [37] have also been explored. Furthermore, MXenes have shown potential applications in polymer composites with significantly improved performances. Ling et al. [38] fabricated exfoliated MXene (Ti_3_C_2_) nanosheets/polyvinyl alcohol (PVA) composite films with high conductivity and flexibility via vacuum filtration. The tensile strength of the composite film with 40 wt.% MXene was about three times larger than that of neat PVA. Zhi et al. prepared Ti_3_C_2_T_x_/polyurethane (PU) nanocomposites via an emulsion method [39]. The results indicated that the addition of 0.5 wt.% MXene increased the yield stress, tensile strength and hardness of PU by approximately 70%, 20% and 13%, respectively. Although MXene-reinforced polymer nanocomposites have been reported, there are still lots of issues to be solved. The hydrophilic exfoliated Ti_3_C_2_ nanosheets are easily oxidized in deionized water in the presence of oxygen. More importantly, exfoliated Ti_3_C_2_ nanosheets cannot be re-dispersed in deionized water or organic solvents after freeze drying. Therefore, the surface modification of exfoliated Ti_3_C_2_ nanosheets using appropriate modifiers for application in polymer nanocomposites is crucial.

Our recent work demonstrated that exfoliated Ti_3_C_2_ nanosheets with a unique 2D structure and transition metal element may serve as a flame retardant and smoke suppression agent for thermoplastic polyurethane elastomer (TPU) [40]. However, the application of Ti_3_C_2_ in flame retarding polystyrene nanocomposites has not been reported yet. In this work, exfoliated Ti_3_C_2_ nanosheets were prepared by etching titanium aluminum carbide (Ti_3_AlC_2_) using lithium fluoride (LiF) plus concentrated hydrochloric acid, followed by ultrasonic treatment in deionized water. These exfoliated Ti_3_C_2_ nanosheets were modified using three kinds of long-chain cationic modifiers, i.e., decyltrimethylammonium bromide (DTAB), octadecyl trimethylammonium bromide (OTAB), and didodecyldimethylammonium bromide (DDAB), and subsequently modified Ti_3_C_2_ nanosheets were incorporated into PS to prepare the nanocomposites. The thermal decomposition, flame retardancy and toxic gas products of the resulting PS nanocomposites were investigated. The flame retardant mechanism was clarified by analyzing the products in condensed and gas phases.

## 2. Materials and Methods

### 2.1. Raw Materials

Polystyrene (PS) with a melt flow rate (25 °C/5 kg) of 3 g/10 min (158 K, density 1.048 g/cm^3^) was provided by Badische Anilin-und-Soda-Fabrik & Yangzi Petrochemical (BASF-YPC) Co. Ltd., Nanjing, China. Ti_3_AlC_2_ was supplied by the Hello Nano Technology Co., Ltd., Changchun, China. Absolute ethanol (AR, 99.7%), *N*,*N*-dimethylformamide (DMF, AR, 99.5%), and hydrochloric acid (HCl, 36.0–38.0 wt.% in H_2_O) were purchased from the Sinopharm Chemical Reagent Co. Ltd., Shanghai, China. DTAB (99%), OTAB (98%), DDAB (97%) and LiF (AR, 99%) were supplied from the Aladdin Reagent Co. Ltd., Shanghai, China. All chemicals were of analytical grade and used as received without further purification.

### 2.2. Preparation of Functionalized MXene Nanosheets

**Scheme 1:**
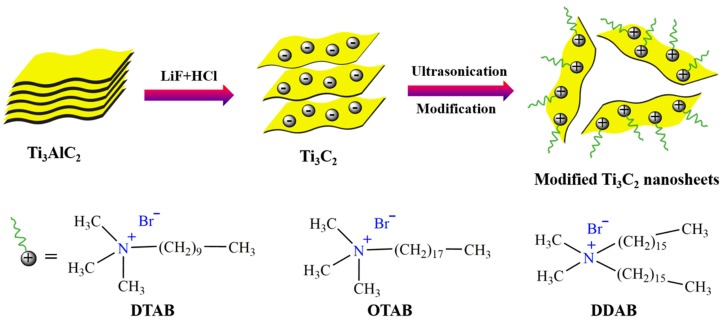
Illustration for preparation of functionalized Ti_3_C_2_ (MXene) nanosheets. DTAB = decyltrimethylammonium bromide; OTAB = octadecyltrimethylammonium bromide; DDAB = dihexadecyldimethylammonium bromide.

Exfoliated Ti_3_C_2_ nanosheets were prepared via etching Ti_3_AlC_2_ in concentrated HCl plus LiF, as described in our recent work [40]. Exfoliated Ti_3_C_2_ nanosheets were modified with cationic modifiers, i.e., DTAB, OTAB and DDAB respectively (Scheme 1), and the detailed process is described as follows. An aqueous suspension of exfoliated Ti_3_C_2_ nanosheets at a concentration of 1 mg/mL was ultrasonicated in an ice bath for 30 min. Then, the cationic modifiers aqueous solution (1 wt.%) was added slowly into the exfoliated Ti_3_C_2_ suspension above at a weight ratio of 0.35/1, and subsequently magnetically stirred for 30 min. Finally, the mixture was centrifuged, and the sediment was washed with deionized water. The resultant product was collected by freeze drying.

### 2.3. Preparation of Functionalized MXene Nanosheets/PS Nanocomposites

Functionalized Ti_3_C_2_ nanosheets/PS nanocomposites were fabricated by a co-coagulation plus compression molding technique. Prior to manufacturing, PS was dried in an oven at 80 °C for 12 h to remove the adsorbed water. Typically, the preparation process of PS/DTAB-Ti_3_C_2_ nanocomposites with 2 wt.% of DTAB-Ti_3_C_2_ is described as follows. DTAB-Ti_3_C_2_ (0.6 g) was dispersed in 250 mL DMF with sonication for 1 h. Subsequently, 29.4 g of PS was introduced into the DTAB-Ti_3_C_2_ dispersion until the formation of a uniform dispersion. Finally, the above solution was poured into deionized water accompanying by magnetic stirring, and subsequently immersed into ethanol. The flocculate obtained was dried at a 60 °C vacuum oven for 12 h to remove residual solvent. The sample was molded using a hot press (YT-LH20D, Yitong Technology Co., Ltd., Dongguan, China) at 195 °C for 10 min with appropriate sizes for further characterizations. PS/OTAB-Ti_3_C_2_ and PS/DDAB-Ti_3_C_2_ nanocomposites were prepared via the same approach. The content of functionalized MXene nanosheets in the three PS nanocomposites was 2 wt.%.

### 2.4. Characterizations

X-ray diffraction (XRD) patterns of samples were recorded on an X-ray diffractometer (Rigaku Co., Tokyo, Japan) with Cu Kα radiation (λ = 0.1542 nm). The morphology of the samples were observed by transmission electron microscopy (TEM) using a JEOL JEM-2100 (Tokyo, Japan) instrument with an acceleration voltage of 200 kV. Prior to observation, power samples were dispersed in deionized water assisted with ultrasonic treatment. Scanning electron microscopy (SEM) was carried out on a Hitachi SU8200 SEM (Tokyo, Japan) with an acceleration voltage of 10 kV. Thermogravimetric analysis (TGA) was carried out on a TGA Q5000IR thermo-analyzer (TA Instruments Inc., New Castle, DE, USA) from 30 °C to 700 °C at a heating rate of 20 °C/min under nitrogen conditions with a flow rate of 6 × 10^−5^ m^3^/min. The weight of all the samples were kept within 5–10 mg. The flame retardant properties of PS and its nanocomposites were evaluated using a cone calorimeter (Fire Testing Technology, Derby, UK) under an incident flux of 35 kW/m^2^. Samples with the dimensions 100 mm × 100 mm × 3 mm were required for the tests. Thermogravimetric analysis/Fourier transform infrared spectrometry (TG-FTIR) of the samples was performed using a TGA Q5000 IR thermogravimetric analyzer that was interfaced to the Nicolet 6700 FTIR spectrophotometer (Thermo Fisher Scientific Inc., Waltham, MA, USA). The sample (5–10 mg) was put in an alumina crucible and heated from 30 °C to 700 °C at a heating rate of 20 °C/min under nitrogen conditions with a flow rate of 6 × 10^−5^ m^3^/min. The stainless steel transfer pipe and gas cell were heated at 230 °C to avoid the condensation of volatile compounds. The FTIR spectra were recorded from 500 cm^−1^ to 4000 cm^−1^. The residues collected in the cone calorimeter tests were analyzed using a SU8010 field-emission scanning electron microscope (FESEM, Tokyo, Japan) coupled with energy dispersive X-ray (EDX). The surface elements were attained from EDX (0.2-20 keV) on an EMAX energy spectroscope (HORIBA, Ltd., Kyoto, Japan). Raman were obtained on a LabRAM-HR Confocal Raman Microprobe (JobinYvon Instruments, Montpellier, France) using a 514.5 nm argon ion laser in the wavenumber range of 100–2000 cm^−1^. All tests for one sample were repeated three times.

## 3. Results and Discussion

### 3.1. Characterizations of Functionalized MXene Nanosheets

**Figure 1:**
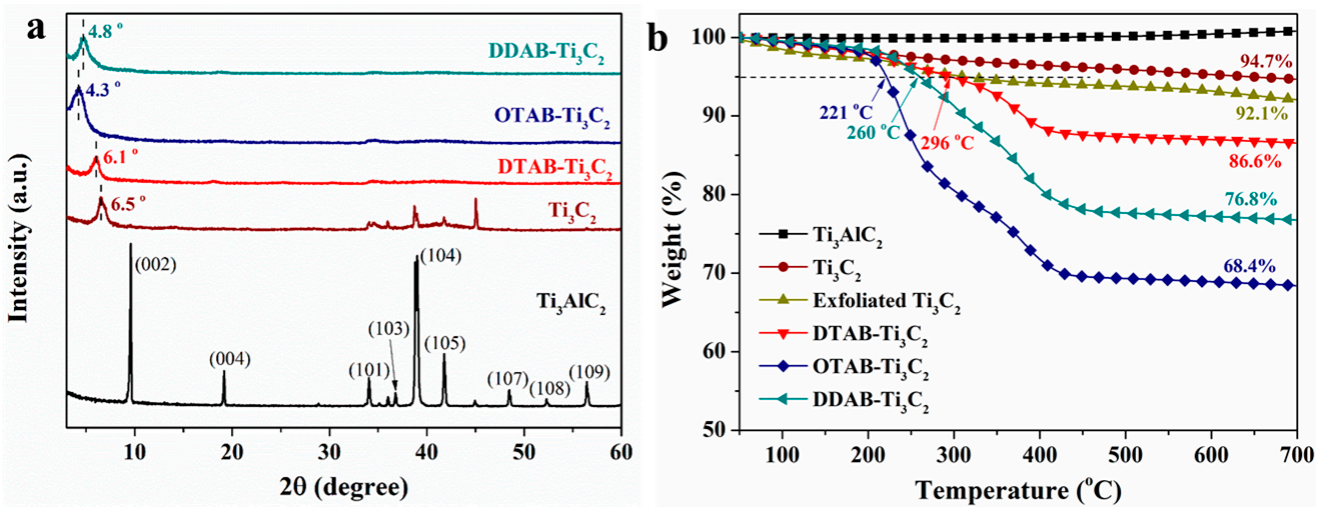
X-ray diffraction (XRD) patterns of Ti_3_AlC_2_, Ti_3_C_2_, DTAB-Ti_3_C_2_, OTAB-Ti_3_C_2_ and DDAB-Ti_3_C_2_; thermogravimetric analysis (TGA) curves of Ti_3_AlC_2_, Ti_3_C_2_, exfoliated Ti_3_C_2_, DTAB-Ti_3_C_2_, OTAB-Ti_3_C_2_ and DDAB-Ti_3_C_2_. a.u. = arbitrary units.

The crystalline phase of bulk Ti_3_AlC_2_, layered Ti_3_C_2_, and the modified Ti_3_C_2_ were studied by XRD and the results are shown in Figure 1a. The bulk Ti_3_AlC_2_ shows characteristic, intense diffraction peaks at 9.6° and 39.0° corresponding to (002) and (104) planes, respectively [41]. However, after successful etching of aluminum in LiF plus concentrated HCl, the intense peak at 39° corresponding to the (104) peak in bulk Ti_3_AlC_2_ disappears completely, which is a clear indication that aluminum has been completely etched from bulk Ti_3_AlC_2_. Moreover, the (002) peak in bulk Ti_3_AlC_2_ shifts to a lower 2θ angle, which suggests an increase in the interlayer distance between the etched Ti_3_C_2_ nanosheets. When etched Ti_3_C_2_ was modified by DTAB, OTAB and DDAB, the interlayer distance between the sheets increased accordingly per the length of the alkyl chains attached for the cationic modifiers respectively. This is evidenced by the reduction in 2θ angle, as shown in Figure 1a. This phenomenon suggests that Ti_3_C_2_ has been successfully modified by the long-chain cationic modifiers. Figure 1b shows the TGA curves of Ti_3_AlC_2_, Ti_3_C_2_, exfoliated Ti_3_C_2_, DTAB-Ti_3_C_2_, OTAB-Ti_3_C_2_ and DDAB-Ti_3_C_2_. Ti_3_AlC_2_ is thermally stable under nitrogen conditions without any weight loss in the temperature range from room temperature to 700 °C. In the high temperature region (approximately 500–700 °C), the weight of Ti_3_AlC_2_ is slightly and gradually increased, which is due to the selective oxidation of aluminum in Ti_3_AlC_2_ upon heating in N_2_ with very low oxygen content [42]. After etching, Ti_3_C_2_ shows gradual mass loss due to the removal of unstable groups, e.g., –F, –OH and –O. Exfoliated Ti_3_C_2_ nanosheets follow a similar degradation process to un-exfoliated Ti_3_C_2_, while the lower residue of 92.1 wt.% at 700 °C is observed, probably due to the presence of tiny amounts of water in the interlayer of Ti_3_C_2_ nanosheets after freeze drying. Modified Ti_3_C_2_ nanosheets exhibit more significant mass loss than exfoliated Ti_3_C_2_ nanosheets, resulting from the grafting of cationic modifiers on their surface. Among modified Ti_3_C_2_ nanosheets, DTAB-Ti_3_C_2_ performs the best, displaying the highest thermal stability and char residues. The initial degradation temperatures at 5 wt.% are 296 °C for DTAB-Ti_3_C_2_, 260 °C for OTAB-Ti_3_C_2_ and 221 °C for DDAB-Ti_3_C_2_, while the corresponding char residues are 86.6%, 76.8% and 68.4% respectively. Based on the char residues, it can be concluded that OTAB is easily attached on the surface of Ti_3_C_2_ nanosheets and OTAB modified Ti_3_C_2_ nanosheets have the worst resistance to thermal degradation.

### 3.2. Morphology and Dispersion

**Figure 2:**
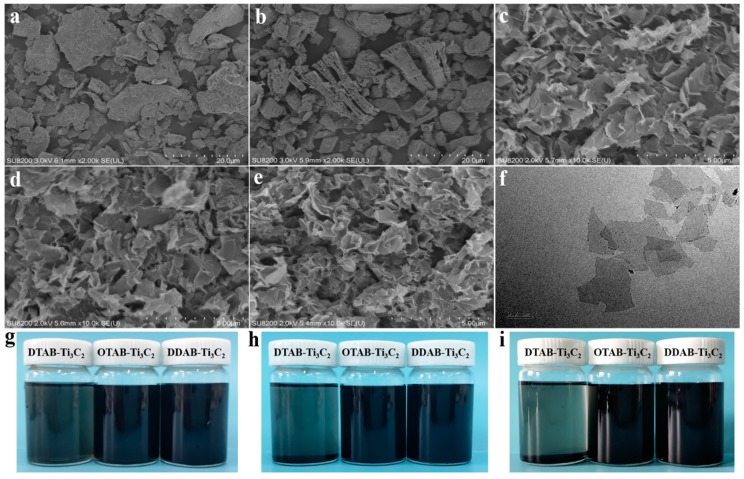
Scanning electron microscopy (SEM) images of (**a**) Ti_3_AlC_2_, (**b**–**e**) etched Ti_3_AlC_2_, DTAB-Ti_3_C_2_, OTAB-Ti_3_C_2_ and DDAB-Ti_3_C_2_; (**f**) transmission electron microscopy (TEM) image of exfoliated Ti_3_C_2_ nanosheets; dispersion of functionalized Ti_3_C_2_ nanosheets in *N*,*N*-dimethylformamide (DMF) for a period: (**g**) 0 h, (**h**) 6 h and (**i**) 24 h.

The morphology of bulk Ti_3_AlC_2_, etched/exfoliated Ti_3_C_2_, DTAB, OTAB, and DDAB modified Ti_3_C_2_ were studied by SEM/TEM, as shown in Figure 2. The SEM micrograph of the bulk Ti_3_AlC_2_ (Figure 2a) before etching shows a characteristic layered structure with high micromechanical cleavage of MAX phases (M, early transition metal; A, A-group element; X, carbon and/or nitrogen) made up of M_n+1_X_n_ piled together in the Al atom layers beside the strong covalent/ionic bonds. However, after the etching and delamination process, a characteristic accordion-like structure with cross-sectional shear slip of weakly stacked multi-layer MXene nanosheets are generated [43]. The individual MXene nanosheets, mainly composed of titanium and carbon, are clearly observed which proves the successful etching of bulk Ti_3_AlC_2_ by the removal of the Al metallic atom layers. From the SEM images (see Figure 2a,b), multiple layers of Ti_3_C_2_ nanosheets are observed with sizes ranging from a single to a several-layered structure compared to the bulk Ti_3_AlC_2_. After modification of Ti_3_C_2_ with DTAB, OTAB and DDAB, a highly dispersed thin layer of nanosheets with a larger interlayer distance can be seen (Figure 2c–e) compared to the unmodified Ti_3_C_2_, which improves the dispersion effect of the various cationic modifiers. The varying alkyl chains with different lengths (which are generally hydrophobic) used in modifying Ti_3_C_2_ can improve the micellar mobility of the nanosheets and prevent it from aggregating. The intercalated long alkyl chains keep the individual Ti_3_C_2_ nanosheets apart preventing them from restacking. This phenomenon is particularly seen in the exfoliated Ti_3_C_2_ modified by cationic agents with double chain alkyl groups (Figure 2e). The long alkyl chains induce weak interlayer bonding between MXene nanosheets, which enhances the dispersion and the formation of irregularly spaced pores that are broadly distributed in a mesoscale. Additionally, the TEM image in Figure 2f shows thin electron beam transparent exfoliated Ti_3_C_2_ nanosheets, thus attesting to the thin nature of the Ti_3_C_2_ nanosheets. To confirm the dispersion ability of modified Ti_3_C_2_ nanosheets, Figure 2g–i shows their dispersion in DMF for a period. Upon ultrasonic treatment, modified Ti_3_C_2_ nanosheets are dispersed in DMF steadily in addition to DTAB-Ti_3_C_2_. After 6 h or 24 h, the uniform dispersion of OTAB-Ti_3_C_2_ and DDAB-Ti_3_C_2_ in DMF is still observed, while DTAB-Ti_3_C_2_ forms a sediment at the bottom of the bottle. This phenomenon can be explained by short chain length and a small amount of DTAB grafted on the surface, as evidenced by the TGA result.

**Figure 3:**
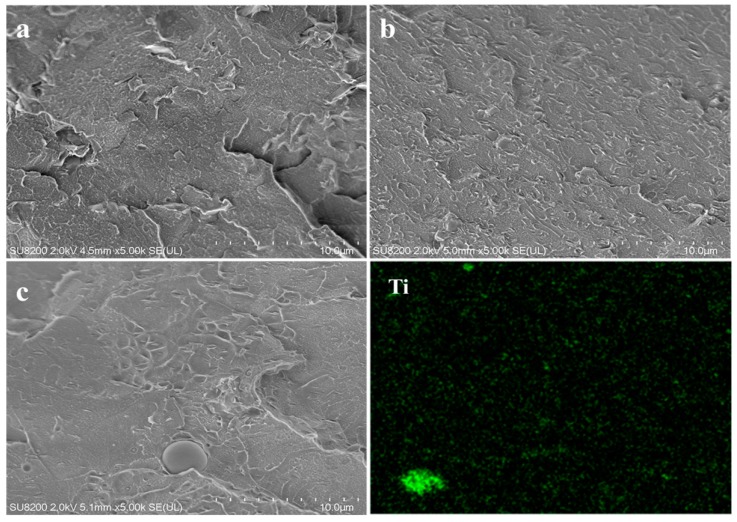
SEM images of the fracture surfaces for (**a**) polystyrene (PS)/DTAB-Ti_3_C_2_, (**b**) PS/OTAB-Ti_3_C_2_, (**c**) PS/DDAB-Ti_3_C_2_ and (**d**) the Ti element mapping of the fracture surface for PS/DDAB-Ti_3_C_2_.

Composites made from high nanofiller loadings (particularly 2D nanomaterials) are often prone to poor filler agglomeration, which results in poor flame retardancy and mechanical properties [44,45]. Therefore, the dispersion of DTAB-Ti_3_C_2_, OTAB-Ti_3_C_2_ and DDAB-Ti_3_C_2_ modified Ti_3_C_2_ in PS was studied by SEM and the results are shown in Figure 3. The fracture surface of DTAB-Ti_3_C_2_/PS composites (Figure 3a) shows no obvious incidence of agglomeration except for what appears to be a few DTAB-Ti_3_C_2_ nanosheets protruding out of the surface. In the OTAB-Ti_3_C_2_/PS composites (Figure 3b), the OTAB modified Ti_3_C_2_ appears uniformly distributed in the polymer matrix with no cluster formation in any spot on the surface. This phenomenon can be attributed to the excellent dispersion enhancement of Ti_3_C_2_ by long chain OTAB, as shown in Figure 2g–i. With the introduction of DDAB modified Ti_3_C_2_ into PS, a few aggregations can be observed besides the uniform dispersion. From the Ti element mapping of the fracture surface for PS/DDAB-Ti_3_C_2_, the Ti is uniformly distributed in the matrix but at the same time aggregates at the bottom center and left side of Figure 3c,d, respectively. This indicates that the Ti_3_C_2_ nanosheets modified with long chain OTAB show the best compatibility with the PS matrix, resulting in superior dispersion.

### 3.3. Thermal Decomposition Behaviors

**Figure 4:**
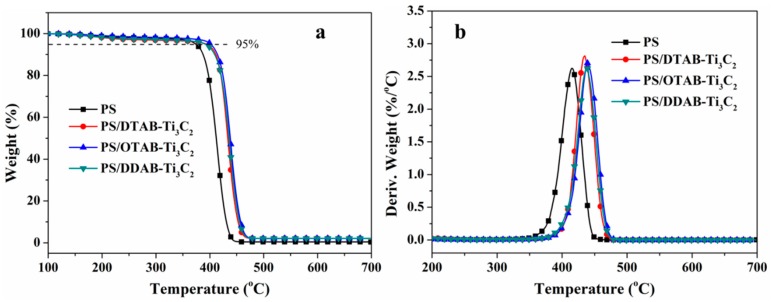
(**a**) TGA and (**b**) derivative thermogravimetry (DTG) curves of PS and its nanocomposites under nitrogen conditions.

The thermal decomposition behaviors of modified Ti_3_C_2_/PS nanocomposites were studied, and the results are shown in Figure 4 and the related data are summarized in Table 1. *T_5%_* is defined as the temperature at 5% weight mass, while *T*_max_ represents the temperature at the maximum degradation rate. PS breaks down in anaerobic conditions by a process of random scissions, yielding products with a lower molecular weight at *T_5%_*. The resulting lower molecular weight fragments get vaporized and diffused out, thus creating the environment for further decomposition. The subsequent major weight loss is due to the rapid and complete depolymerization of PS chains which results in the formation of char (0.4 wt.%). The addition of DTAB-Ti_3_C_2_, OTAB-Ti_3_C_2_ and DDAB-Ti_3_C_2_ into PS result in no obvious changes in the decomposition trend/rate of PS before reaching *T_5%_*, although an increase at *T_5%_* is observed (see Table 1). However, from the mass change rate (derived from the TGA curve), modified Ti_3_C_2_ leads to the slight improvement of maximum mass decomposition compared to pristine PS, although *T*_max_ values increase (see Figure 4b). The relatively higher mass loss at the maximum decomposition temperature by the modified Ti_3_C_2_/PS is due to early degradation of surface termination groups of Ti_3_C_2_ (i.e., –F, –O, –OH, and Li) induced by the etching process and subsequent degradation by the alkyl chains of the cationic modifiers combined with the catalytic effect of Ti_3_C_2_ nanosheets [40]. Nevertheless, the cationic modified Ti_3_C_2_ is thermally stable and enhanced the charring ability of PS. PS/OTAB-Ti_3_C_2_ has the highest thermal stability but the char yield remains the same as DTAB and DDAB modified Ti_3_C_2_/PS composites. The char residues which are rapidly generated on the surface of the polymer matrix act as a shield to isolate the polymer from heat and air [46,47,48]. Therefore, the improvement in char yield is beneficial for protecting the underlying polymer matrix against the supply of volatile pyrolysis products and oxygen, therefore reducing the propensity for further fire propagation.

### 3.4. Flame Retardant Properties

**Figure 5:**
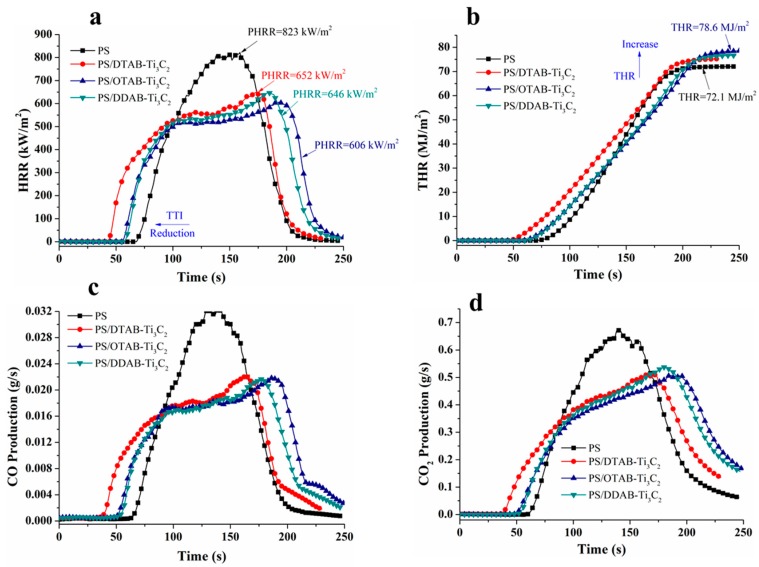
(**a**) HRR, (**b**) total heat release (THR), (**c**) CO and (**d**) CO_2_ production curves of PS and its nanocomposites. PHRR = peak heat release rate.

The combustion behaviors of the various cationic modified Ti_3_C_2_/PS nanocomposites were measured via cone calorimeter due to its ability to acquire information about flammability, smoke and toxic fumes production [49,50,51]. The flame retardant properties of PS and its nanocomposites were measured by cone calorimeter and the results are shown in Figure 5 and Table 2. The PHRR of pristine PS is high at about 823 kW/m^2^ compared to those of the flame retarded composites. The PHRR values of the composites are reduced considerably as shown in Figure 5a, with OTAB-Ti_3_C_2_/PS having the lowest PHRR (approximately a 26.4% reduction), probably due to the better dispersion of OTAB-Ti_3_C_2_ in PS than the other two counterparts. This attests to the improvement in flame retardancy of PS/MXene nanocomposites. Meanwhile, the total heat release (THR) values for the nanocomposites are slightly higher than pristine PS due to the relatively broad PHRR peak area resulting from the prolonged combustion, as shown in Figure 5b. The toxic gases released during the combustion of the composites were measured and the results are shown in Figure 5c–d. Similar to the combustion phenomenon of PHRR, the peak CO production (PCOP) for PS/DTAB-Ti_3_C_2,_ PS/OTAB-Ti_3_C_2,_ and PS/DDAB-Ti_3_C_2_ are generally lower by approximately 31.7%, 32.3% and 32.9% respectively, but unlike the PHRR, the DDAB-Ti_3_C_2_/PS composite has the lowest PCOP value. Figure 5d shows the peak CO_2_ production (PCO_2_P) during the entire combustion period. The CO_2_ production rate follows the similar trend as the PHRR because CO_2_ production (CO_2_P) relates to the rate of conversion of the partial oxidation product to the full oxidation products/residues. The results indicate that cationic modified Ti_3_C_2_ can effectively inhibit toxic fume release during the combustion process of PS nanocomposites. The toxic fume reduction mechanism of DTAB, OTAB, and DDAB modified Ti_3_C_2_/PS nanocomposite is based on the tortuous path and heat sink effect of MXene nanosheets and various types of TiO_2_ formed during the combustion process. The tortuous path effect is very plausible due to the high aspect ratio of Ti_3_C_2_ nanosheets and the fact that it has been uniformly dispersed within the PS matrix. The combined heat sink and tortuous path effect slows down the exchange of gases in the flame zone, thereby reducing the burning rate and enhancing the fire safety of PS.

### 3.5. Volatile Product Analysis

**Figure 6:**
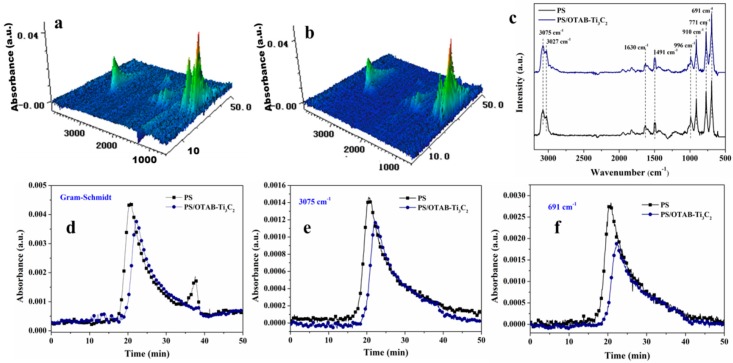
3D TG-FTIR spectra of (**a**) PS and (**b**) PS/OTAB-Ti_3_C_2_; (**c**) FTIR spectra of volatile products of PS and PS/OTAB-Ti_3_C_2_ at maximum degradation rate; Gram–Schmidt curves of total absorbance (**d**), hydrocarbon compounds (**e**) and aliphatic compounds (**f**).

The volatile pyrolysis products that evolved during the thermal degradation of pristine PS and cationic modified Ti_3_C_2_/PS composites were studied by TG-FTIR under a nitrogen atmosphere and the results are shown in Figure 6. The 3D TG-FTIR spectra of the volatile pyrolysis products of PS and OTAB-Ti_3_C_2_ obtained at the onset of decomposition (365 °C) and maximum decomposition (416 °C) are shown in Figure 6a–b, with both polymers showing similar pyrolysis products. PS begins to decompose at 365 °C into smaller styrene oligomers, dimers, and trimer unites around 3090 cm^−1^, 3020 cm^−1^, 1600 cm^−1^, 1491 cm^−1^ and 698 cm^−1^, with the absorptions becoming much stronger at *T_max_* (416 °C). Similar decomposition peaks can be identified in the OTAB-Ti_3_C_2_/PS composites, except with relatively low intensity due to the presence of OTAB-Ti_3_C_2_ in PS. Figure 6c shows the FTIR spectra of volatile pyrolysis products of PS/OTAB-Ti_3_C_2_ obtained at *T*_max_. Peaks similar to the 3D TG-FTIR are evolved around 3075 cm^−1^, 3027 cm^−1^, 1630 cm^−1^, 1491 cm^−1^, 996 cm^−1^, 910 cm^−1^, 771 cm^−1^, and 691 cm^−1^, which are assigned to the degradation products of the styrene oligomer. No obvious additional peaks can be seen in PS/OTAB-Ti_3_C_2_, indicating that similar peaks are evolved by pristine PS and its flame retarded composites. During thermal degradation, PS undergoes random scission of chains to form one primary radical and one secondary benzyl radical with a strong benzylic resonance, during which minimal gases are formed. As the temperature increases, chain-end scission, intermolecular abstractions (the radicals abstract the hydrogen from a different molecule or the surrounding environment), recombination reactions and disproportionation reactions of radicals occur in the pyrolysis process, during which more volatiles evolve [52]. The Gram–Schmidt curves of aromatic hydrocarbons and aliphatic compounds evolved during the pyrolysis process are shown in Figure 6d–f. Obvious reductions are observed for the PS/OTAB-Ti_3_C_2_ nanocomposite compared to pristine PS, which reinforces the pyrolysis products blocking effect by the tortuous path effect.

### 3.6. Residue Analysis

**Figure 7:**
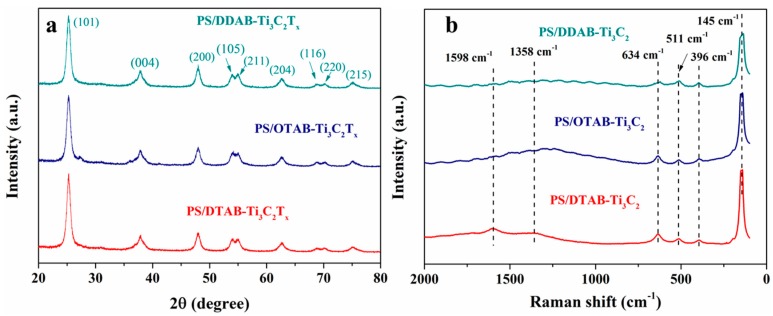
XRD patterns and Raman spectra of the char residues for PS/DTAB-Ti_3_C_2_, PS/OTAB-Ti_3_C_2_ and PS/DDAB-Ti_3_C_2_.

**Figure 8:**
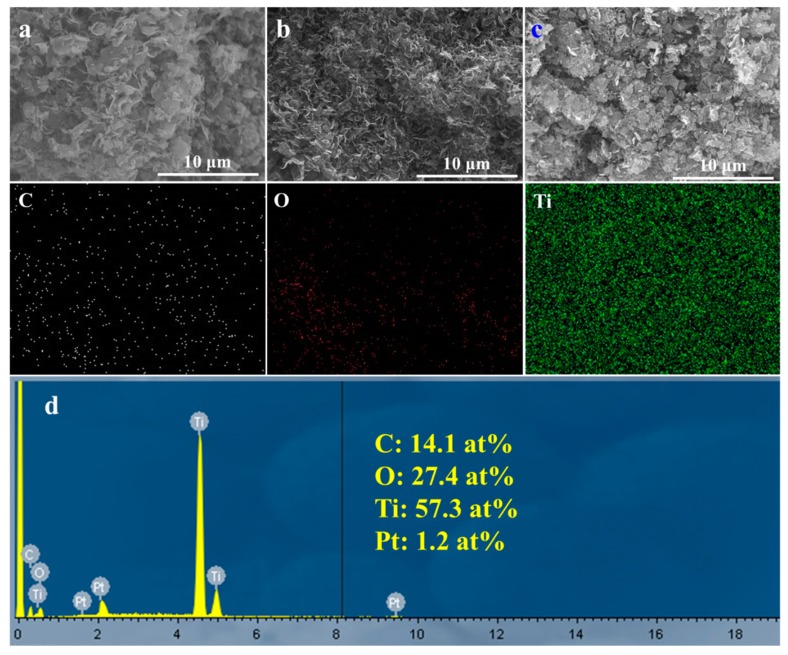
SEM images of the char residues for (**a**) PS/DTAB-Ti_3_C_2_, (**b**) PS/OTAB-Ti_3_C_2_ and (**c**) PS/DDAB-Ti_3_C_2_; (**d**) energy dispersive X-ray (EDX) spectrum and atomic percentage of the residue for PS/OTAB-Ti_3_C_2_.

To confirm the chemical composition and structure of the residual chars, XRD was performed and Raman spectra were taken, and the results are shown in Figure 7a–b. The XRD pattern shown in Figure 7a conforms to the anatase phase of titanium dioxide, similar to reference [53]. From the Raman spectra, the amorphous and graphitic carbon peaks around 1358 cm^−1^ and 1598 cm^−1^ are almost missing, especially in PS/OTAB-Ti_3_C_2_ and PS/DDAB-Ti_3_C_2_ residual chars, as confirmed by the SEM/EDS images in Figure 8. However, peaks belonging to the various phases of TiO_2_ can be found in the Raman spectra around 634 cm^−1^, 511 cm^−1^, 396 cm^−1^ and 145 cm^−1^ [54]. This indicates that the residual char resulting from the cone calorimeter test is largely made up of the anatase phase of TiO_2_ with few carbons. Therefore, modified Ti_3_C_2_ nanosheets undergo thermal oxidation during burning, leading to the formation of TiO_2_ as observed in prior work [55].

The char residue generated after the cone calorimeter test was studied by SEM/EDX to understand the flame-retardant mechanism of the cationic modified Ti_3_C_2_/PS composites and the results are shown in Figure 8a–d. All the char residues of PS/DTAB-Ti_3_C_2_, PS/OTAB-Ti_3_C_2_, and PS/DDAB-Ti_3_C_2_ show nanosheet morphology which is similar with the dispersed structure shown in Figure 2c–e. The char structure is attributed mainly to the formation of TiO_2_ nanosheets after combustion, as confirmed by XRD and Raman results. The EDX of the PS/OTAB-Ti_3_C_2_ nanocomposite is shown in Figure 8d. As indicated, a large proportion of the char residue is made of the anatase phase of TiO_2_, and oxygen and carbon are represented in comparatively small proportions of approximately 27.4% and 14.1% respectively. The MXene nanosheets together with the TiO_2_ nanosheets in the char can act as a heat sink during the combustion process and create a physical barrier to reduce the degradation of PS.

### 3.7. Comparison of Thermal Stability and Flame Retardancy

To highlight the progress in thermal stability and flame-retardant properties of PS/MXene, the comparison of PS and its 2D nanomaterials-based composites in this work to the results reported in previous literature are summarized in Table 3. Zhou et al. reported that graphene nanosheets (GNS) [28] or cetyltrimethyl ammonium bromide (CTAB)-modified MoS_2_ [56] showed a small reduction in PHRR. Unfortunately, CTAB-modified MoS_2_ resulted in serious deterioration of the thermal stability of PS. Bao et al. [57] enhanced the fire-retardant performance of PS by introducing phosphazene-decorated graphene oxide (GO). However, the nanocomposites exhibited reduced thermal stability. Other 2D nanomaterials, such as organic-modified layered zirconium phosphate (OZrP, Zhang et al. [58]), layered zirconium phosphate (Tai et al. [1]) and layered double hydroxide (Matusinovic et al. [59]) were also not effective to reduce the PHRR, but simultaneously reduced the thermal stability of PS. In our previous study, a polyaniline (PANI)/thermal-exfoliated hexagonal boron nitride (BNO) hierarchical structure (PANI-BNO) was prepared via an in-situ deposition approach [60]. Compared to pure PS, PS/PANI-BNO showed enhanced thermal stability and fire safety properties. In the current work, both the thermal stability and flame-retardant performance of PS-based nanocomposites were considerably improved when functionalized MXene nanosheets were added into the PS matrix. More importantly, the loading of functionalized MXene for PS was lower than that in the reported articles, indicating the remarkable reinforcement effect in thermal stability and fire safety properties.

## 4. Conclusions

In this work, exfoliated MXene nanosheets were successfully modified by long-chain cationic agents, i.e., DTAB, OTAB and DDAB to enhance its dispersion and interfacial adhesion in PS and simultaneously improve the thermal stability and flame retardancy, and reduce the production of toxic fumes. The modified MXene nanosheets were well dispersed in DMF before compounding with PS. The thermal stability of cationic modified Ti_3_C_2_/PS nanocomposites improved considerably. The surface modification of Ti_3_C_2_ with the cationic modifiers increased the interlayer distance between the MXene nanosheets and enhanced its dispersion and interface interaction within PS matrix. The modified MXene nanosheets improved the flame-retardant properties of PS by significantly reducing the PHRR by approximately 20.8% for PS/DTAB-Ti_3_C_2_, 21.5% for PS/DDAB-Ti_3_C_2_ and 26.4% for PS/OTAB-Ti_3_C_2_. Significant reductions in CO and CO_2_ productions were also obtained and the lower release of pyrolysis volatile products was recorded for PS/OTAB-Ti_3_C_2_ compared to pristine PS. The shielding barrier and catalytic effect of modified Ti_3_C_2_ nanosheets and the subsequently formed TiO_2_ nanosheets were responsible for the improved flame retardancy and suppressed toxic fumes. This work will provide a simple approach to surface modification of MXenes for promising applications in flame retardant polymer nanocomposites.

## Figures and Tables

**Table 1:** TGA data of PS and PS nanocomposites under nitrogen conditions (20 °C/min, 5–10 mg; errors ± 0.5 wt%, ± 1 °C). *T_5%_* = the temperature at 5% weight mass; *T*_max_ = the temperature at the maximum degradation rate.

Sample No.	*T*_5%_ (°C)	*T*_max_ (°C)	Char Residues (700 °C, wt%)
Neat PS	374	415	0.4
PS/DTAB-Ti_3_C_2_	394	435	2.1
PS/OTAB-Ti_3_C_2_	399	440	2.1
PS/DDAB-Ti_3_C_2_	397	438	2.1

**Table 2:** Cone calorimeter data for each sample at 35 kW/m^2^. (TTI: time to ignition; PHRR: peak heat release rate; THR: total heat release; PCO_2_P: peak CO_2_ production; PCOP: peak CO production).

Sample No.	TTI (s)	PHRR (kW/m^2^)	THR (MJ/m^2^)	PCOP (g/s)	PCO_2_P (g/s)
Error	±2	±15	±0.5	±0.005	±0.02
Neat PS	62	823	72.1	0.0322	0.673
PS/DTAB-Ti_3_C_2_	52	652	75.2	0.0220	0.511
PS/OTAB-Ti_3_C_2_	49	606	78.6	0.0218	0.507
PS/DDAB-Ti_3_C_2_	35	646	76.6	0.0216	0.537

**Table 3:** Thermal stability and flame retardant performance of PS nanocomposites reported in previous work and this work (CC: cone calorimeter; MCC: micro-combustion calorimeter).

Sample No.	Additives Loading (wt.%)	*T_5%_* (°C)	PHRR (kW/m^2^)/Technique	Ref.
PS/GNS	3	+13	−17.4%/CC	[28]
PS/CTAB-MoS_2_	3	−114	−9.6%/MCC	[56]
PS/FGO	3		−52.7%/CC	[57]
PS/OZrP	5	−73		[58]
Poly(St-co-AEPPA)/ZrP	3	−17	−13.9%/MCC	[1]
PS/CaAl LDH-B	3		−19.1%/CC	[59]
PS/PANI-BNO	3	+5	−31.3%/CC	[60]
PS/DTAB-Ti_3_C_2_	2	+20	−20.8%/CC	This work
PS/OTAB-Ti_3_C_2_	2	+25	−26.4%/CC	This work
PS/DDAB-Ti_3_C_2_	2	+23	−21.5%/CC	This work
